# Association of Dietary Habits and Interest for Food and Science versus Weight Status in Children Aged 8 to 18 Years

**DOI:** 10.1155/2018/4061385

**Published:** 2018-01-21

**Authors:** Els Vanderhulst, Aicha Faik, Johan Vansintejan, Inès Van Rossem, Dirk Devroey

**Affiliations:** Department of Family Medicine and Chronic Care, Vrije Universiteit Brussel, Brussels, Belgium

## Abstract

**Introduction:**

This study aims to describe the association between dietary habits and weight status and the interest in food and science.

**Methods:**

We examined in a cross-sectional study 525 children aged between 8 and 18 years, who attended the Brussels Food Fair or the Belgian Science Day in 2013. They were divided into three groups: special interest in science, special interest in food, and a general control group. They completed a questionnaire, and body parameters were measured. The weight status of the children was identified using the growth charts and the calculated BMI.

**Results:**

In total, 525 children were included: 290 children in the reference group, 194 in the food group, and 41 in the science group. The prevalence of overweight and obesity was 28% in the general control group, 14% in the food group, and 15% in the science group. Breakfast and dinner were skipped more often by children with overweight or obesity. Children from the food and science groups had more sweets and meat, had less fruit, and skipped less meals.

**Conclusion:**

In our study, 28% of the reference group had overweight or obesity. The children with special interest in food or science differed from the control group.

## 1. Introduction

According to the World Health Organization (WHO), overweight and obesity take worldwide epidemic proportions in recent decades and form a serious health problem for the population [1]. This trend is observed in adults, children, and adolescents [1–3].

In 2015, more than 100 million children were estimated to be obese. Over the past 25 years, the prevalence of obesity in children has increased in almost all countries, and it doubled in 70 countries. In most developed countries, the increase of obesity is greater in children than that in adults [4].

Overweight and obesity are associated with comorbidities such as hypertension, dyslipidemia, diabetes, arthritis, sleep apnea, and others. Thereupon, there is an association with some psychosocial consequences such as psychological distress, anxiety, depressive disorders, low self-esteem, and eating disorders [3–7]. Even more alarming is the fact that children who are overweight or obese have a higher risk to become obese adults [2–4, 6, 8, 9].

### 1.1. Global Epidemic

In Belgium, the number of children with overweight and obesity doubled since the eighties [9, 10]. The most recent Belgian Health Interview Survey in 2013 estimated the prevalence of overweight and obesity in Belgian children between 2 and 17 years at 20.0% and 7.1%, respectively, whereas in 1997, only at 14.9% and 4.9%, respectively [11].

Another study showed that 26% of Belgian children were overweight, of which 8% were obese. In 1997, these rates were, respectively, only 21% and 5%, showing the rapid increase of the problem [10]. Belgium follows the trends in other European countries and the Western world [10].

### 1.2. Causes of Obesity

Obesity often results from a complex interaction between lifestyle and genetic factors. Especially in childhood, the role of genes in obesity is important. In the near future, it will be possible to detect the genes responsible for obesity in childhood and to start an early treatment [12]. Several publications confirm an association between overweight and obesity on one hand, and bad nutritional habits and a lack of physical activity on the other. This means that obesity is mainly due to an increased supply of dietary energy and a reduced dissipation of energy due to low physical activity [3, 5, 7].

However, other factors may also be of influence. Several authors mention the regularly skipping of breakfast as another risk factor for the development of overweight and obesity [6, 13]. Keast et al. mentioned conflicting statements in the literature on the association between obesity and eating snacks between the three main meals [7]. Fabry et al. suggested in 1964 that a more frequent food intake would be inversely proportional to the body weight [14]. Booth et al., on the other hand, described that eating more than three meals a day would contribute to the development of obesity [15]. Finally, there was a consensus that there was less overweight among children who had two or more snacks per day [7].

### 1.3. Aim of the Study

Firstly, this study aims to investigate the association between weight status and dietary habits such as snacking, drinking soft drinks, having breakfast, having a lunch and a dinner, eating vegetables, fruits, meat, and bread, and drinking milk in Belgian children aged 8 to 18 years.

Secondly, we compare dietary habits and weight status of children interested in food or science with a general control group of children visiting the fair with their school classes. Preliminary results were presented at the 21st WONCA World Conference of Family Doctors [16]. The association of weight and factors such as physical activity and interest in food and sciences was already reported in a previous publication [17].

## 2. Methods

### 2.1. Study Population

This cross-sectional study was conducted at the Brussels Food Fair from October 5 to 20, 2013, and at the National Science Day on November 24, 2013. A total of 525 children were enrolled during these two occasions.

Three study groups were defined. The first study group was a general control group of children between 8 and 18 years of age, visiting the fair with their school classes. They were recruited during class visits of schools who organized a trip to the Brussels Food Fair.

Twenty-five schools were invited: 10 French-speaking schools and 15 Dutch-speaking schools corresponding to the language distribution in Belgium. Sixteen of 25 invited schools visited the fair. Students belonged to classes from the second and third cycles of primary school (8 to 12 years of age) (*n* = 4), as well as to classes from general secondary school (*n* = 4), technical secondary education (*n* = 4), vocational technical education (*n* = 3), and art secondary education (*n* = 1). Only 3% of the parents of invited children refused to sign the informed consent, and another 4% forgot to sign it.

The second group were children with special interest in food. They were recruited during the weekends among the children who visited the Brussels Food Fair with their parents.

The third group were children with special interest in science, who were recruited during the National Science Day. During that yearly event, scientific institutes and universities demonstrate scientific experiments on different locations to make children familiar with scientific research. The accompanying parents received at both occasions a brief explanation about the design of the study and the investigated parameters.

For the three groups, the parents had to give their informed consent, prior to participation. For the control group, the parents signed the informed consent at their home, and the children brought the document to the fair as the parents did not accompany the children. For the food and science groups, the parents accompanied the children, and they signed the document at the fair at the moment of participation.

### 2.2. Inclusion and Exclusion Criteria

Inclusion criteria for this study were being aged between 8 and 18 years of age and having an informed consent signed by one of the parents. Exclusion criteria were pregnancy and showing visible signs of alcohol, drug, or medication abuse. The researchers were trained to detect signs of alcohol, drug, or medication abuse. Female participants aged 14 years or older were asked about pregnancy. None of the participants were excluded due to this.

### 2.3. Questionnaire

The study consisted in part of completing a standardized questionnaire developed by the Netherlands Nutrition Centre to identify the dietary habits of the participants (Supplementary Materials (available here)) [18]. This questionnaire was completed at the fair with the neutral assistance of one of the researchers. The children were asked if they had some sweets or soft drinks the day before the fair, if they had breakfast, lunch, and dinner, if they ate fruits, vegetables, meat, and bread, and if they drank milk.

### 2.4. Physical Examination

Weight, height, waist circumference, and blood pressure were measured at the fair for all participants. For the measurements, we used a weighing scale (Seca Sensa 804), a measuring stick (Seca Body Meter 206), a measuring tape, a sphygmomanometer (Welch Allyn DS54), and a stethoscope (Littmann Classic II SE), respectively.

After the examination, the children received a personalized advice based on the answers they gave to the questionnaire, their eating habits, and body mass index (BMI). The determination of normal BMI values was based on the Flemish growth curves of the Vrije Universiteit Brussel [19]. On these curves, the calculated BMI values were plotted as a function of the sex and age of the children studied. If the BMI values were located on these curves in the grey zone between percentiles 85 and 97, participants were classified as overweight. BMI values above percentile 97 corresponded to the category of obesity. The upper and lower limits of that grey zone correspond to respective BMI values of 25 and 30 at the age of 18.

### 2.5. Approval Ethical Committee

For this study, the approval of the Ethics Committee of the University Hospital Brussels was obtained.

### 2.6. Statistical Analyses

Statistical analyses were carried out using IBM^®^ SPSS^®^ Statistics 22. Participant data which were incomplete with regard to weight and height were excluded for the analyses. Two-by-two tables were used to evaluate, by means of a chi-square test, the relationship between two dichotomous variables. The Fisher exact test was used when at least one expected value was below five. The *T*-test was used to compare the means of two independent samples. All study results were standardized for age and gender by direct standardization according to the Belgian population aged between 8 and 18 years of 2013.

## 3. Results

### 3.1. Demographics

The total study population consisted of 525 children from 8 to 18 years of age (42% boys and 58% girls): 290 children in the reference group, 194 in the food group, and 41 in the science group (Table 1). The children originated from 167 different municipalities. The average age and the median age were both 11 years. There was no significant difference for age in the three study groups, but in the science group, the boys were overrepresented.

### 3.2. Body Mass Index

Two-thirds of the total study population had a normal BMI, 10% was underweight, 17% was overweight, and 5% was obese (Table 2). There were no significant differences between girls and boys.

Only one participant had severe underweight (Table 3). Not less than 6.6% of the reference group had underweight. In the food and science groups, the proportion of children with underweight was almost twice as high (*p*(2)=0.035). In the reference group, 28% of children were overweight or had obesity. In the food and science groups, the proportion of children with overweight or obesity was half as high (*p*(2)=0.001).

### 3.3. Dietary Habits

Not less than 10% of the participants skipped their breakfast the day before the study, and 5% had skipped their dinner (Table 4). Sweets and soft drinks were taken by 46% and 57%, respectively. Essential dietary products such as milk and fruit were skipped by 69% and 70%, respectively. Girls were taking more sweets than boys (*p*=0.004), and boys used more milk or yogurt than girls (*p*=0.002).

The proportions of children skipping their meals was higher in the group with overweight or obesity, as compared to the group with normal weight or underweight (Table 5). Especially breakfast and dinner were skipped by the group with overweight or obesity. Surprisingly, the group with normal weight was eating more meat and sweets.

Only 34% of the participants of the reference group reported to have had a sweet the day before the study (Table 6). These proportions were significantly higher in the food and science groups (*p*(2) < 0.001). On the contrary, only 37% of the children of the science group declared to have had a soft drink the day before. This proportion was significantly higher in the reference group and the food group (*p*(2)=0.005). Children from the reference group skipped more meals, especially breakfast (*p*(2)=0.047) and supper (*p*(2) < 0.001).

The children from the food and science groups had more fruit (*p*(2) ≤ 0.001) and more meat (*p*(2) ≤ 0.001). There were no significant differences between the groups in respect to vegetables, bread, and milk or yogurt.

## 4. Discussion

### 4.1. Prevalence of Overweight and Obesity

This study showed that more than one-fifth of the total study population had overweight or obesity. When taking into account the general control group, the proportion of children with overweight or obesity is 28%. Not less than 20% of the children had overweight and 8% was obese. This corresponds good with the most recent Belgian Health Interview Survey of 2013 that estimated the prevalence of overweight and obesity in Belgian children between 2 and 17 years at 20.0% and 7.1%, respectively [11].

A similar survey in 1997 showed that 21% of Belgian children had overweight and that 5% of them showed obesity [12]. A 2001 study found that 26% of the Belgian children had overweight and that 8% was obese [10]. This study suggested an increase of the prevalence of overweight in the coming decades.

Taking into account the worldwide epidemic of obesity, we expected at least an increase of the proportions of children with obesity in our study. A possible explanation for the fact that we did not find an increase is that selection bias occurred in our research. The children and their parents were asked if they agreed to participate in the study. This was maybe refused more often by children who knew they had a weight disorder.

### 4.2. Dietary Habits

It is at least remarkable that children with overweight or obesity report to have less sweets than children without overweight. This would mean that the intake of more sweets is associated with a lower body weight. This striking conclusion has already been done by Keast et al. [7] in 2010 and by Fabry et al. [14] in 1964. This could be explained by the fact that children who were aware of their obesity either ate less sweets, unconsciously forgot to report the sweets for different reasons, or have not responded honestly to the question. These possible explanations were also cited by Keast et al. [7]. Another hypothesis could be that a higher energy intake may be compensated with more physical activity. However, these conclusions are not supported by our study because the data were not available.

With regard to breakfast, our results are similar to the work of Croezen et al. [6] and Szajewska and Ruszczynski [13]. Children who are overweight or obese skip breakfast more often than children without weight problems. Thereupon, not having dinner is also associated with overweight in our study, and for supper, the *p* value was also almost significant. As a result, our study suggests that skipping one of the meals is a marker for a lifestyle that leads to overweight. It was already known that the regularly skipping of breakfast is a risk factor for the development of overweight and obesity [6, 13]. But our observation that skipping one of the meals is related to overweight is rather new.

Another remarkable observation is that children without overweight report to eat meat more frequently than children with overweight. It is difficult to put this observation in the right perspective, without having a detailed dietary analysis. Maybe some children using processed fast food such as hamburgers and other fried meats did not report them as “meat” in the questionnaire.

About one-third of the children in the three groups had a weight disorder. Whereas in the general control group, most of the children with a weight disorder were overweight and only 7% had underweight. This was different in the food and science groups. The proportion of children with underweight in these groups was almost as high as the proportion of children with overweight.

The association between the skipping of meals and overweight is the most relevant clinical finding from our study. Earlier research came to a similar conclusion but only for breakfast [6, 13]. Our study suggests that skipping any meal may be a marker for a lifestyle that leads to overweight. Although we could not demonstrate a causal link, it seems plausible to advice children with (a risk for) overweight not to skip meals and to adopt a healthy lifestyle.

### 4.3. Limitations of the Study

The cross-sectional nature of the study did not allow to provide answers on the casualty of overweight and obesity.

As mentioned above, there was possibly a selection bias for overweight or obese children who chose not to participate in the study, in contrast to children who had a healthy lifestyle. We tried to minimize this selection bias by inviting complete classes, hoping that all children of the classes participated.

This study did not take into account the financial and socioeconomic situation of the parents. They could have had an influence on the access to healthy food and explain some differences between groups. Since children usually do not know the financial situation of their parents sufficiently for this kind of analysis, these questions could have been given to parents when they signed the informed consent. However, this was not done to avoid parents not signing the informed consent because they felt restraint to communicate their financial situation. Moreover, British researchers concluded that the purchase of healthy food was not necessarily expensive [20].

The second group were children with special interest in food or at least their parents had special interest in food. However, it was not clear whether they were interested in healthy food or in food in general.

The fact that participants for the food and science groups were only recruited in the weekend is another limitation of the study. The chance to have breakfast is possibly higher during weekends than on weekdays. However, this was partly remediated by the fact that children included on Saturdays (food and science groups) reported on their diet on Fridays and children included on Mondays (reference group) reported on their diet on Sundays.

A similar issue can be made for the intake of sweets and fruit. The chance to eat a piece of fruit is higher during the week, certainly in schools with a policy on healthy food. On the other hand, it is possible that children with a healthy lifestyle during weekdays are only allowed to have sweets in the weekends.

Finally, some prudence is necessary in generalizing the results of the control group to the general pediatric population. Although the children of the control group were recruited in schools without a special interest in food, there still might be some bias because the children were enrolled at a food fair.

### 4.4. Strengths of the Study

This was a large study with 525 participants. The children originated from at least 167 different municipalities, which guarantees a fair distribution of the population over the 559 Belgian municipalities. Another advantage of this study was that immediately after the investigation, the children received a personalized advice based on their dietary habits and BMI. In this way, the study created some awareness about unhealthy dietary habits.

### 4.5. Future Research

The biometric data such as weight and height are collected on a regular basis through the Pupil Guidance Centres. The continuous monitoring and analyses of these figures will reduce selection bias and give more representative results. The visits to these centres could also be a good opportunity to let children complete a more comprehensive questionnaire on lifestyle and dietary habits, for example, over a period of one week before the visit to the centre. This would give a better analysis of the average diet of the children than a sample of a single day in the week. The visit to the Pupil Guidance Centres could also give the opportunity to give a personalized health and dietary advice.

## 5. Conclusions

Not less than one-third of the children in the study population did not had a normal weight. Most children suffered from overweight and obesity, but in the groups with special interest in food or science, the proportion of children with underweight was also important. Children from the food and science groups had more sweets and meat, but they had less fruit and skipped less meals. Children with overweight or obesity skipped breakfast or dinner more often.

## Figures and Tables

**Table 1:** Demographics for the three study groups.

	Total study population (*n* = 525)	Reference group (*n* = 290)	Food group (*n* = 194)	Science group (*n* = 41)
Number	525	290	194	41
Boys (%)	223 (42%)	128 (44%)	71 (37%)	24 (59%)
Girls (%)	302 (58%)	162 (56%)	123 (63%)	17 (41%)
Mean age (*y*)	11	11	12	10
Median age (*y*)	11	10	12	10

**Table 2:** Distribution of girls and boys according to the BMI classes.

	Total study population (*n* = 525)	Boys (*n* = 223)	Girls (*n* = 302)	*p* value
Underweight	9.7%	8.5%	10.6%	0.501
Normal weight	68.4%	71.3%	66.2%	0.216
Overweight	16.6%	15.2%	17.5%	0.483
Obesity	5.3%	4.9%	5.6%	0.726

Percentiles at the age of 18 years: underweight = BMI < 18.4; normal weight = 18.5 < BMI < 24.9; overweight = 25.0 < BMI < 29.9; obesity ≥ 30.

**Table 3:** Body mass index groups for the reference group and the food and science groups.

	Total study population (*n* = 525)	Reference group (*n* = 290)	Food group (*n* = 194)	Science group (*n* = 41)	*p*(2) value
Underweight	9.7%	6.9%	13.4%	12.2%	0.035
Normal weight	68.4%	65.2%	72.2%	73.2%	0.21
Overweight	16.6%	20.3%	11.3%	14.6%	0.031
Obesity	5.3%	7.6%	3.1%	0.0%	0.028
Obesity and overweight	21.9%	27.9%	14.4%	14.6%	0.001

Percentiles at the age of 18 years: underweight = BMI < 18.4; normal weight = 18.5 < BMI < 24.9; overweight = 25.0 < BMI < 29.9; obesity ≥ 30.

**Table 4:** Comparison of nutritional habits of girls and boys.

	Total (*n* = 525)	Boys (*n* = 223)	Girls (*n* = 302)	*p* value
Sweets	46%	39%	52%	0.004
Soft drinks	57%	59%	55%	0.39
Breakfast	90%	91%	88%	0.33
Dinner	95%	93%	96%	0.11
Supper	94%	93%	94%	0.84
Vegetables	81%	83%	79%	0.38
Fruit	70%	70%	69%	0.71
Meat	74%	73%	74%	0.85
Bread	87%	85%	89%	0.18
Milk or yogurt	69%	77%	64%	0.002

**Table 5:** Comparison of nutritional habits for normal weight and overweight.

	Overweight or obesity (*n* = 115)	Normal weight or underweight (*n* = 410)	*p* value
Sweets	37%	49%	0.017
Soft drinks	55%	57%	0.66
Breakfast	83%	91%	0.006
Dinner	89%	96%	0.001
Supper	90%	95%	0.051
Vegetables	82%	80%	0.76
Fruit	65%	71%	0.26
Meat	59%	78%	<0.001
Bread	85%	88%	0.51
Milk	72%	71%	0.46

**Table 6:** Comparison of nutritional habits for the reference group and the food and science groups.

	Total study population (*n* = 525)	Reference group (*n* = 290)	Food group (*n* = 194)	Science group (*n* = 41)	*p*(2) value
Sweets	46%	34%	62%	59%	<0.001
Soft drinks	57%	55%	63%	37%	0.005
Breakfast	90%	87%	93%	93%	0.047
Dinner	95%	93%	97%	95%	0.19
Supper	94%	89%	99%	100%	<0.001
Vegetables	81%	80%	81%	83%	0.92
Fruit	70%	77%	60%	63%	<0.001
Meat	74%	66%	85%	78%	<0.001
Bread	87%	85%	88%	98%	0.083
Milk or yogurt	69%	71%	67%	71%	0.78

## Supplementary Materials

Supplementary Materials The supplementary material provides the standardized questionnaire, developed by the Netherlands Nutrition Centre, that was completed to identify the dietary habits of the participants.
