# Supplementary material for: Association of Dietary Habits and Interest for Food and Science versus Weight Status in Children Aged 8 to 18 Years
Source: J Obes. 2018 Jan 21;2018:4061385. doi: 10.1155/2018/4061385 (PMC5828495; doi:10.1155/2018/4061385)
Supplement: Supplementary Materials — The supplementary material provides the standardized questionnaire, developed by the Netherlands Nutrition Centre, that was completed to identify the dietary habits of the participants. [file 4061385.f1.pdf]

Supplementary material

Questionnaire

What is your gender?	O Man	O Woman
What is your zipcode?	_____	
What is your year of birth?	_____	

How would you rate your health on a scale from 1 to 100 where 1 corresponds to "very poor" and 100 "very good"? Put a cross on the line.

1---100

Weight:	____kg
Height:	____cm
Abdominal circumference:	____cm
Blood pressure:	____/____mmHg

Are you affiliated with a sports club?	O Yes	O No	O Don't know
Did you have some sweets yesterday?	O Yes	O No	O Don't know
Did you have some soft drinks yesterday?	O Yes	O No	O Don't know
Did you have breakfast yesterday?	O Yes	O No	O Don't know
Did you have dinner yesterday?	O Yes	O No	O Don't know
Did you have supper yesterday?	O Yes	O No	O Don't know
Did you have some vegetables yesterday?	O Yes	O No	O Don't know
Did you have some fruit yesterday?	O Yes	O No	O Don't know
Did you have some meat yesterday?	O Yes	O No	O Don't know
Did you have some bread yesterday?	O Yes	O No	O Don't know
Did you have some milk or yoghurt yesterday?	O Yes	O No	O Don't know
How do you usually go to school?	O By bus or car	O On foot or by bicycle	
